# Investigation of BRCAness associated miRNA-gene axes in breast cancer: cell-free miR-182-5p as a potential expression signature of BRCAness

**DOI:** 10.1186/s12885-022-09761-4

**Published:** 2022-06-17

**Authors:** Farzaneh Darbeheshti, Sepideh Kadkhoda, Mahsa Keshavarz-Fathi, Sepideh Razi, Afshin Bahramy, Yaser Mansoori, Nima Rezaei

**Affiliations:** 1 Department of Medical Genetics, School of Medicine, Tehran University of Medical Sciences, Tehran, Iran; 2 Cancer Immunology Project (CIP), Universal Scientific Education and Research Network (USERN), Tehran, Iran; 3 School of Medicine, Tehran University of Medical Sciences, Tehran, Iran; 4 School of Medicine, Iran University of Medical Sciences, Tehran, Iran; 5 Department of Medical Genetics, Faculty of Medical Sciences, Tarbiat Modares University, Tehran, Iran; 6 Noncommunicable Disease Research Center, Fasa University of Medical Sciences, Fasa, Iran; 7 Department of Medical Genetics, Fasa University of Medical Sciences, Fasa, Iran; 8 Research Center for Immunodeficiencies, Children’s Medical Center, Tehran University of Medical Sciences, Dr. Qarib St, Keshavarz Blvd, Tehran, Iran; 9 Department of Immunology, School of Medicine, Tehran University of Medical Sciences, Tehran, Iran; 10 Network of Immunity in Infection, Malignancy and Autoimmunity (NIIMA), Universal Scientific Education and Research Network (USERN), Tehran, Iran

**Keywords:** Breast cancer, Triple-negative, BRCAness, miRNAs, Cell-free biomarker

## Abstract

The concept of the ‘BRCAness’ phenotype implies the properties that some sporadic breast cancers (BC) share with *BRCA1*/*2*-mutation carriers with hereditary BC. Breast tumors with BRCAness have deficiencies in homologous recombination repair (HRR), like *BRCA1*/*2*-mutation carriers, and consequently could benefit from poly-(ADP)-ribose polymerase (PARP) inhibitors and DNA-damaging chemotherapy. Triple-negative breast cancers (TNBC) show a higher frequency of BRCAness than the other BC subtypes. Therefore, looking for BRCAness-related biomarkers could improve personalized management of TNBC patients. microRNAs (miRNAs) play a pivotal role in onco-transcriptomic profiles of tumor cells besides their suitable features as molecular biomarkers. The current study aims to evaluate the expression level of some critical miRNAs-mRNA axes in HRR pathway in tumors and plasma samples from BC patients. The expression levels of three multi-target miRNAs, including miR-182-5p, miR-146a-5p, and miR-498, as well as six downstream HRR-related protein-coding genes, have been investigated in the breast tumors and paired adjacent normal tissues by Real-time PCR. In the next step, based on the results derived from the previous step, we examined the level of cell-free miR-182-5p in the blood plasma samples from the patients. Our results highlight the difference between TNBC and non-TNBC tumor subgroups regarding the dysregulation of the key miRNA/mRNA axes involved in the HRR pathway. Also, for the first time, we show that the level of cell-free miR-182-5p in plasma samples from BC patients could be a clue for screening BC patients eligible for receiving PARP inhibitors through a personalized manner. Altogether, some sporadic BC patients, especially sporadic TNBC, have epigenetically dysregulated HRR pathway that could be identified and benefit from BRCAness-specific therapeutic agents.

**Supplementary Information:**

The online version contains supplementary material available at 10.1186/s12885-022-09761-4.

## Background

Breast cancer (BC) is the most common cancer and the leading cause of cancer-related death among women [1]. Gene expression profiling research has identified at least four subtypes of breast cancer, including luminal A, luminal B, HER2 positive, and triple-negative [2]. Triple-negative tumors are more aggressive than other subtypes and are associated with a poorer prognosis. Moreover, patients with triple-negative breast cancer (TNBC) do not respond to hormonal therapies and HER2-targeted therapies like Herceptin [3, 4].

Familial breast cancer accounts for only 5–10% of all breast cancer cases and is mainly caused by germline mutations in BRCA1, BRCA2, ATM, BRIP1, CHEK2, TP53, and PTEN genes [5]. Most of these genes are involved in DNA repair pathways. Homologous recombination DNA repair (HRR) is a process that repairs DNA double-strand breaks (DSBs) with a minimum probability of errors. Several factors such as a mutation in BRCA1 or BRCA2 gene lead to homologous recombination deficiency (HRD), which results in activation of alternative repairing pathways, including non-homologous end joining (NHEJ) that is an error-prone DNA repair pathway [6]. Interestingly, defect in the HRR pathway in patients with sporadic breast cancer can mimic the phenotype of familial BRCA1 and BRCA2 mutation carriers which is termed as BRCAness. Also, studies have reported that the majority of the triple-negative breast tumors show BRCAness [7]. In patients with sporadic breast cancer, defect in the HRR system can be caused by somatic mutations or alterations in the gene expression of the HRR pathway members. The oral poly (ADP-ribose) polymerase (PARP) inhibitors such as Olaparib and Talazoparib have been approved as monotherapies for BRCA-mutated metastatic BC with HRD [8–10].

Some studies have evaluated PARP inhibitors and DNA-damaging chemotherapy for the treatment of somatic TNBC [11]. It seems that these therapeutic strategies have potential benefits for patients with BRCAness phenotype who have HRR pathway deficiency. In recent years, researchers have looked for the BRCAness footprint in DNA alterations by high throughput techniques such as next-generation sequencing (NGS), array comparative genomic hybridization (aCGH) [12], single-nucleotide polymorphism (SNP) assay [13], and multiplex ligation-dependent probe amplification (MLPA) [9]. In 2017, it was reported that whole genome sequencing (WGS) could identify HR-deficient tumors based on somatic mutational signatures including indels, base substitutions, copy number variations, and rearrangements. These mutational signatures are associated with *BRCA1*/*2* dysfunction because of either somatic mutations or functional deficiency without detectable mutations. This WGS-based predictor called HRDetect and is capable to distinguish *BRCA1*/*2*-deficient samples with high sensitivity [14].

However, it is well known that epigenetic mechanisms could change protein expression without any changes in DNA sequences [15, 16]. RNA interference, as an epigenetic mechanism, has prominent roles in the dysregulation of gene expression profile in tumor cells [17, 18].

MicroRNAs (miRNAs) are a subset of small non-coding RNAs which could post-transcriptionally regulate the gene expression. They target the 3′ untranslated region (UTR) of target mRNAs, leading to mRNAs degradation or translation inhibition depending on the complementarity between the miRNA and target mRNA. In the last decade, miRNA expression profiling showed that miRNAs could be used for tumor identification, classification, diagnosis, prognosis, and treatment. Moreover, cell-free miRNAs in biofluids are extremely stable in adverse conditions such as high and low PH, boiling, and frequent freeze-thaw cycles. It has been shown that miRNAs have the potential to be considered novel non-invasive biomarkers for cancer managment [19, 20].

In this study, as BRCAness phenotype is important in BC management, we have investigated the expression of some critical miRNAs-mRNA axes in the HRR pathway in BC samples. Also, cell-free miR-182 expression, the most dysregulated miRNA in the breast tumors, was evaluated in plasma samples from the patients.

## Methods

### GEO microarray profiling data and literature review

**Fig. 1:**
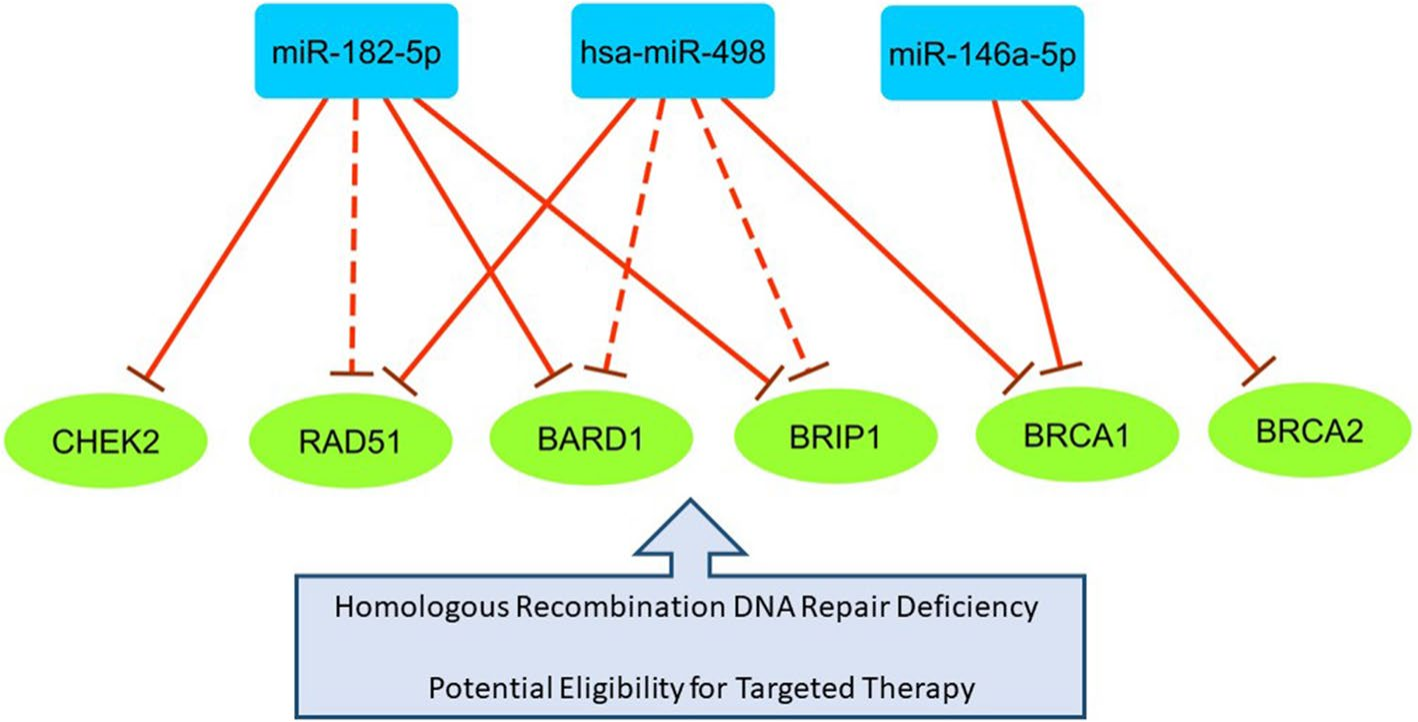
The miRNA/mRNA regulatory axes involving in the HRR pathway in BC. The blue rectangles and green circles represent microRNAs and mRNAs, respectively. The solid lines show experimentally validated interactions, and the dash lines represent bioinformatically predicted interactions

To uncover the expression signatures of BRCAness phenotype in BC patients, in the first step, we screened the differentially expressed (DE) miRNAs in BC based on GEO microarray dataset GSE19536, GPL8227 (Supplementary Table 1). This dataset totally consists of 101 tumor samples, including 48 luminal A, 19 luminal B, 21 TNBC, and 13 HER2^+^ subtypes). In the next step, we sought DE miRNAs that potentially target multiple essential genes involved in HRR pathway according to Targetscan, DIANA, and miRTarBase databases, besides the literature review. We considered the following criteria to select miRNAs in the current study. 1) Showing a significant upregulation in TNBC based on the microarray data; 2) Targeting ≥2 genes that are involved in HRR pathway, and their interactions have been experimentally confirmed. Accordingly, three multi-target miRNAs, including miR-182-5p, miR-146a-5p, and miR-498 have been picked out from the DE miRNAs. Moreover, the six protein-coding genes that play pivotal roles in HR DNA repair have been considered as miRNAs’ targets (Fig. 1). The conditional selection of miRNAs’ targets is based on the above-mentioned databases and breast cancer literature review [21–23].

The expression levels of all Fig. 1 compartments have been evaluated in the tumor and adjacent normal samples. In addition, their expression differences have also been determined between TNBC and non-TNBC tumors because of the high frequency of BRCAness phenotype in TNBC. Finally, the expression correlation matrix among them has been illustrated.

### Participants

The sixty-two Iranian women with sporadic breast cancer, including 32 TNBC and 30 non-TNBC, referred to Shahid Faghihi Hospital, Shiraz, Iran, participated in this study. These women had not received chemotherapy, radiotherapy, and immunotherapy before surgery. Sixty-two paired tissues (tumor and adjacent normal samples that have been pathologically approved) and 53 plasma samples were obtained from the patients. Plasma samples from ten healthy volunteer women were also obtained to calculate the relative expression. The current study was approved by the Ethics committee of Tehran University of Medical Sciences (TUMS) (Code of Ethics: IR.TUMS.CHMC.REC.1398.137). It is confirmed that all experiments were performed in accordance with relevant guidelines and regulations. Moreover, the written consents were assigned by all participants. The clinical features, including age at diagnosis, tumor size, ER/PR/HER2 status, grade, and lymph node metastasis, were achieved from the hospital records.

### RNA extraction

Total RNA was extracted from three samples of breast tumors, paired adjacent normal tissues, as well as from plasma samples with TRIzol reagent (Invitrogen, USA) following the manufacturer’s protocol. The RNA isolation from plasma samples was optimized according to Petra Cerkovnik et al. reports [24]. The quality and integrity of the extracted RNA were measured by Nanodrop 2000C (Thermo Scientific, USA) and 1.5% or 2% agarose gel electrophoresis, respectively. Afterward, the cDNA was synthesized using the PrimeScriptTM 1st strand cDNA Synthesis Kit (TaKaRa Bio, Japan) by the mixture of oligo-dT and random hexamer primers. The cDNA synthesis for miR-182-5p, miR-146a-5p, miR-498, and RNU44 was performed using specific stem loop RT primers (Table 1).

**Table 1:** The primer sequences which used in RT-PCR and quantitative real-time PCR

Primer name	Primer Sequence (5′ → 3′)
**miR-182-5p**	RT: GTCGTATCCAGTGCAGGGTCCGAGGTATTCGCACTGGATACGACAGTGTGAG
	F: GTTTGGCAATGGTAGAACTCACAC
	R: GTGCAGGGTCCGAGGT
**miR-146a-5p**	RT: GTCGTATCCAGTGCAGGGTCCGAGGTATTCGCACTGGATACGACAACCCA
	F: CGAACTGAGAACTGAATTCCA
	R: GTGCAGGGTCCGAGGT
**miR-498**	RT: GTCGTATCCAGTGCAGGGTCCGAGGTATTCGCACTGGATACGACGAAAAA
	F: TATATTTCAAGCCAGGGGGCG
	R: GTGCAGGGTCCGAGGT
**BRCA1**	F: CCCTCAAGGAACCAGGGATG
	R: GCTGCACGCTTCTCAGTGGT
**BRCA2**	F: AGACTGTACTTCAGGGCCGTACA
	R: GGCTGAGACAGGTGTGGAAACA
**BRIP1**	F: CAACTATCCAAGCACACCACC
	R: AACTTTGCAGCCAGAGTGGTT
**BARB1**	F: ACAACTGGACAGCATGATTCAAC
	R: ACTTCGAGGGCTAAACCACA
**RAD51**	F: CCAGACCCAGCTCCTTTATC
	R: CACTGCTACACCAAACTCATC
**CHEK2**	F: TCAGCAAGAGAGGCAGACCC
	R: ACAGCTCTCCCCCTTCCATC
**PUM1**	F: AGTGGGGGACTAGGCGTTAG
	R: GTTTTCATCACTGTCTGCATCC
**RNU44**	RT: GTCGTATCCAGTGCAGGGTCCGAGGTATTCGCACTGGATACGACAGTCAGTT
	F: GAATGCTGACTGAACATGAAGGTC
	R: GTGCAGGGTCCGAGGT

### Quantitative real-time PCR

The relative expression of miRNAs and mRNAs in all samples was evaluated using RealQ Plus 2× Master Mix Green low ROX (Ampliqon, Denmark) by the LightCycler 96 (Roche, Germany) in duplicate. PUM1 and RNU44 housekeeping genes were applied to normalized data derived from the genes and miRNAs, respectively. Base on the previous reports, these two genes are two of the most stable houskeeping genes among breast normal and tumor tissues [25]. The reaction conditions for all genes, except miR-498, consist of 15 minutes preincubation at 95 °C followed by amplification in 40 cycles in 95 °C for 15 seconds and then 60 °C for 45 seconds. The annealing temperature for miR-498 was 62 °C. The sequence of primers and PCR product lengths are listed in Table 1. The RT primers for miRNAs were designed using stem loop sequence 5′-GTCGTATCCAGTGCAGGGTCCGAGGTATTCGCACTGGATACGAC-3′. For mRNAs, one of the primers (forward or reverse) spans an exon-exon junction to avoid DNA amplification in the Real-time PCR reactions. The relative expression of RNAs (fold change) was calculated by 2^−ΔΔCt^method (ΔΔCT = ΔCT_target_ ‐ ΔCT_calibrator_).

### Statistical analysis

The data were analyzed and visualized using SPSS Statistics software v.26 and GraphPad Prism v.8. The normality of data was evaluated by Kolmogorov-Smirnov test. The paired sample t-test and paired samples Wilcoxon test were applied to compare the differences between the means of tumor and adjacent non-tumorous samples with normal and non-normal distribution, respectively. For comparison of gene expression between the TNBC and non-TNBC samples, an unpaired sample t-test or Mann-Whitney test was used. Spearman correlation statistic was run to assess the expression correlation between the miRNAs and their targets. We have applied the unpaired sample t-test or ANOVA for normal data and Mann-Whitney or Kruskal-Wallis test for data with non-normal distribution to investigate the relationship between gene expression and patients’ clinicopathological features.

## Results

### Evaluating the expression differences of miR-182-5p, miR-146a-5p, and miR-498 between breast tumors and adjacent normal tissues

**Fig. 2:**
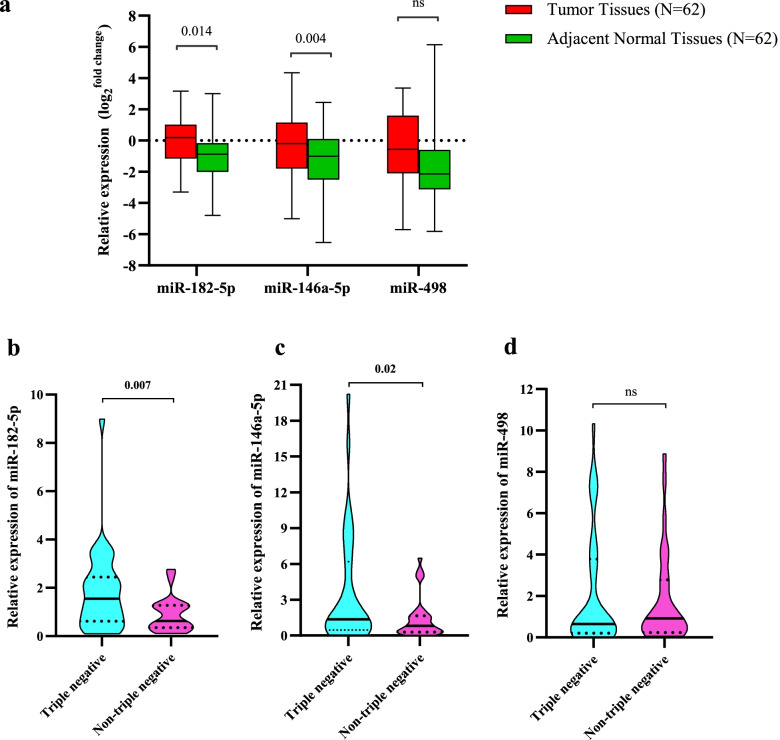
**a** Expression levels of miR-182-5p, miR-146a-5p, and miR-498 in breast tumors and matched adjacent normal tissues. **b** Comparing the expression levels of miR-182-5p between TNBC and non-TNBC subtypes. **c** Comparing the expression levels of miR-146a-5p between TNBC and non-TNBC subtypes. **d** Comparing the expression levels of miR-498 between TNBC and non-TNBC subtypes

The miR-182-5p and miR-146a-5p show a significant up-regulation in the breast tumors compared with the paired adjacent normal tissues (Fig. 2a). The P.value of expression differences for the former miRNA is 0.014 and for the latter one is 0.004. The mean of miR-498 expression in breast tumors is higher than adjacent normal tissues. Although, this difference does not reach statistical significance (Fig. 2a).

### Investigating the expression levels of miR-182-5p, miR-146a-5p, and miR-498 between triple-negative breast cancer (TNBC) and non-TNBC subtypes

In order to evaluate the likely differences of expression profiles of miR-182-5p, miR-146a-5p, and miR-498 between TNBC and non-TNBC tumor subtypes, we have compared their relative expression between these two groups. Interestingly, the expression levels of miR-182-5p and miR-146a-5p were significantly higher in TNBC tumors in comparison with non-TNBC ones (Fig. 2b and c). Concerning miR-498, our result indicated that there is not any significant difference in its expression between the two groups (Fig. 2d).

### Evaluating the expression differences of BRCA1, BRCA2, CHEK2, BRIP1, RAD51, and BARD1 between breast tumors and adjacent normal tissues

**Fig. 3:**
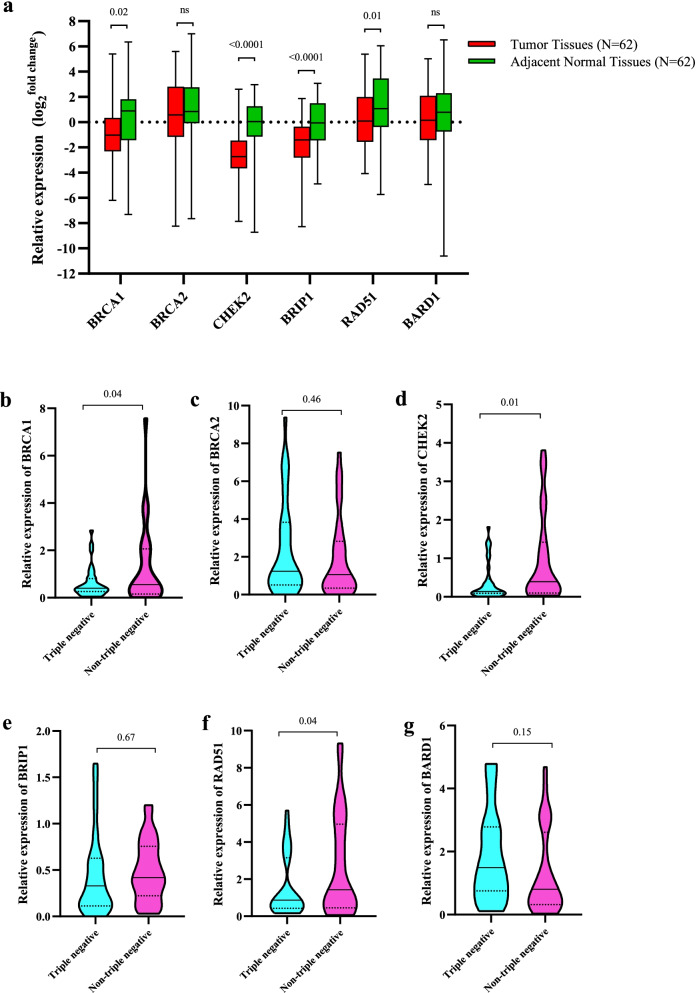
**a** Expression levels of BRCA1, BRCA2, CHEK2, RAD51, BARD1, and BRIP1 in breast tumors and matched adjacent normal tissues. **b-g** Comparing the expression levels of BRCA1 (b), BRCA2 (c), CHEK2 (d), BRIP1 (e), RAD51 (f), and BARD1 (g), and between TNBC and non-TNBC subtypes

We have selected the six essential genes in the HR DNA repair pathway, which are potentially located downstream of the miRNAs mentioned above as their targets. The gene expression investigations revealed the substantial down-regulation of BRCA1 (*P* = 0.02), CHEK2 (*P* = < 0.0001), BRIP1 (*P* = < 0.0001), and RAD51 (*P* = 0.01) in breast tumors compared with adjacent normal tissues (Fig. 3a). However, BRCA2 and BARD1 did not show any significant dysregulation in the breast tumors.

### Investigating the expression levels of BRCA1, BRCA2, CHEK2, BRIP1, RAD51, and BARD1 between triple-negative breast cancer (TNBC) and non-TNBC subtypes

The expression differences of six essential genes in the HR DNA repair pathway were examined between TNBC and non-TNBC tumors. This survey indicates BRCA1, CHEK2, and RAD51 have significantly lower expression in TNBC in comparison with non-TNBC (Fig. 3b, d, and f). The three genes BRCA2, BRIP1, and BARD1, did not reveal any significant differences between the two breast tumor subtypes (Fig. 3c, e, and g).

### Exploring the expression correlation of the miRNA-gene axes in the breast normal and tumor samples

**Fig. 4:**
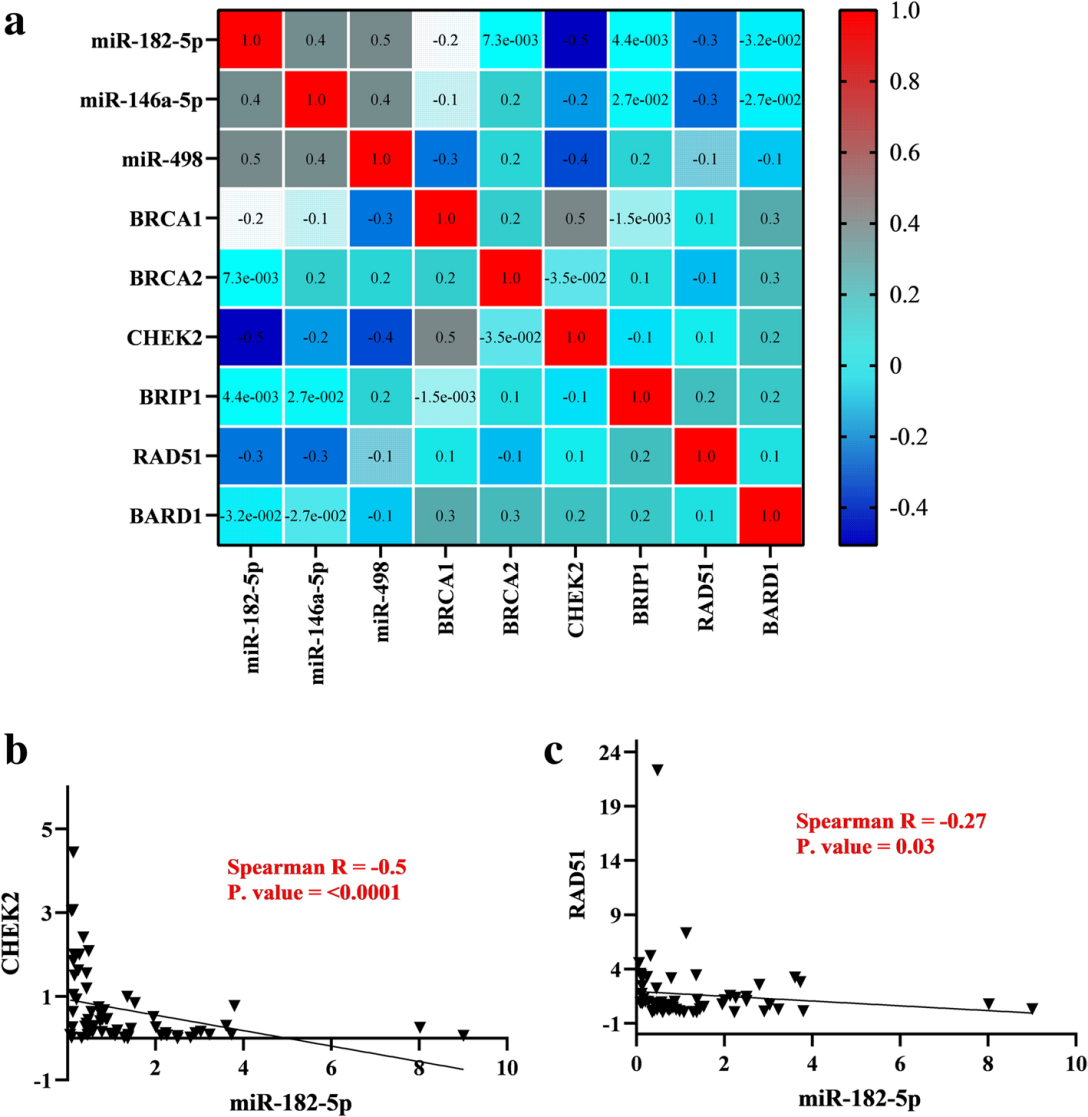
**a** The expression correlation matrix between studied miRNAs (miR-182-5p, miR-146a-5p, and miR-498) and mRNAs (BRCA1, BRCA2, CHEK2, RAD51, BARD1, and BRIP1) among 62 breast tumor samples has been represented by a color scale based on Spearman’s correlation coefficient (R). Darker blue colors represent a stronger negative correlation and darker red colors show a stronger positive correlation. **b** The correlation analyses of miR-182-5p expression level with CHEK2 among 62 tumor samples. **c** The correlation analyses of miR-182-5p expression level with RAD51 among 62 tumor samples

Spearman correlation test was run to assess the expression correlation between compartments of the miRNA-gene axes in Fig. 1, and the expression correlation matrix among them has been illustrated (Fig. 4a). Among all potential miRNA-mRNA pairs which is depicted in the figure, only miR-182-5p show significant negative correlation with its two downstream targets, CHEK2 (r = − 0.5, *P* = < 0.001, Fig. 4b), RAD51 (r = − 0.27, *P* = 0.03, Fig. 4c). The linear regression analyses indicate the significant linear effect of miR-182-5p expression on down regulating CHEK2, RAD51 (Supplementary Table 2). It revealed that every 0.3 unit decrease in miR-182-5p expression could increase CHEK2 and BRIP1 expression by 1.4 units. Considering miR-182-5p/RAD51 axis, every 1.8 unit decrease in miR-182-5p expression could have a linear impact on increasing RAD51 expression by 7.8 unit.

The two other miRNAs, miR-146a-5p and miR-498, mostly show negative correlations with their potential targets (Supplementary Tables 3 and 4). However, these negative correlations did not meet the cutoff of statistically significant.

The heatmap from the expression data shows the expression patterns of the studied transcripts across all the tumor and normal tissues (Supplementary Fig. 1).

### Investigation of cell-free miR-182-5p in the blood plasma samples from breast cancer patients

**Fig. 5:**
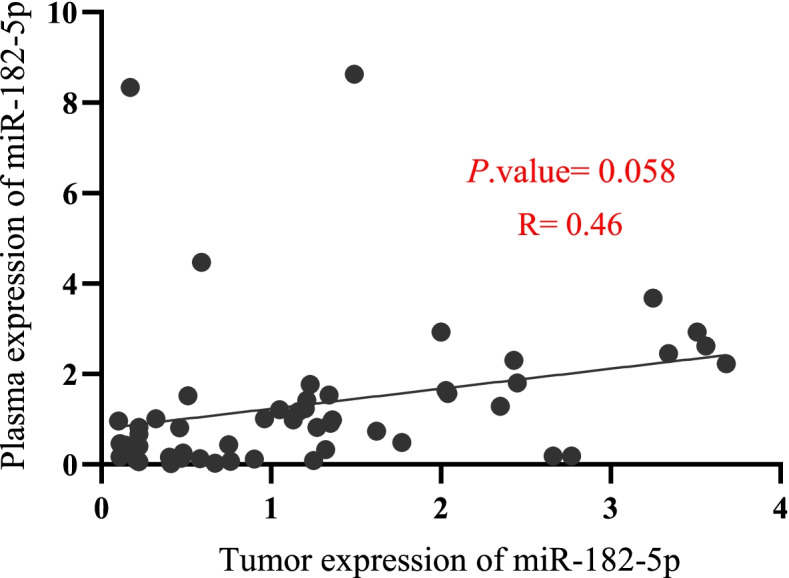
Correlation analysis of miR-182-5p expression between tumor and plasma samples from fifty-three BC patients; R: Pearson’s correlation coefficient

Since our data about miR-182-5p expression indicate its remarkable up expression in breast tumors, especially in TNBC and its potential effects on downregulating CHEK2 and RAD51 in TNBC, we examined the level of cell-free miR-182-5p in the blood plasma samples from the patients. Interestingly, a positive correlation has been detected between its levels in the patients’ tumor and plasma samples (r = 0.46, *P* = 0.058, Fig. 5).

To investigate the expression status of cell-free miR-182-5p in the patients, we categorized its expression into three groups: over-expression, normal expression, and under-expression. As the expression levels of cell-free miR-182-5p in ten healthy controls were between 0.09 to 2.13, the values 2.15 or more were included in the over-expression group, and values 0.07 or less were considered to belong to the under-expression group. We have shown the frequency of patients in each group in Table 2. This analysis reveals that 32% of TNBC patients and only 7.2% of non-TNBC patients show an up-regulated level of cell-free miR-182-5p in their plasma samples.

**Table 2:** Compring the expression status of cell-free miR-182-5p between plasma samples from TNBC and non-TNBC patients

Cell free expression of miR-182-5p	Non-triple negative (***n*** = 28)	Triple negative (***n*** = 25)	Total plasma samples (***n*** = 53)
**Mean of relative expression level (range)**	1.06 (0.02–8.34)	1.66 (0.07–8.63)	1.34 (0.02–8.63)
**Expression status**			
Under expression (%)	3 (10.7)	0	3 (5.6)
Normal expression (%)	23 (82.1)	17 (68)	40 (75.4)
Over expression (%)	2 (7.2)	8 (32)	10 (19)

### Correlation between miR-182-5p expression level and the clinicopathological features

The higher expression of miR-182-5p in the breast tumors reveals a significant association with larger tumors, higher tumor grade, and positive lymph nodes (Table 3). The association studies between clinicopathological variables of patients and all studied transcripts have been summarized in Table 3.

**Table 3:** Association of the transcripts expression levels with clinicopathological features in BC patients

Characteristic	Subgroups	^a^miR-182-5p	miR-146a-5p	miR-498	BRCA1	BRCA2	CHEK2	BRIP1	RAD51	BARD1
Age	< 50 (32)	1.08	0.94	0.95	0.52	1.11	0.12	0.36	0.83	1.02
	≥50 (30)	1.18	1.16	0.64	0.45	1.29	0.44	0.39	1.06	1.2
	*P*.value	0.75	0.79	0.23	0.93	0.47	**0.03**	0.74	0.9	0.98
Tumor size	< 2 (16)	0.53	0.68	0.39	0.3	0.63	0.77	0.36	0.62	0.84
	2–5 (40)	1.18	1.21	0.77	0.49	1.57	0.24	0.44	1.18	1.57
	> 5 (6)	2.63	1.02	0.74	0.46	0.88	0.12	0.25	0.94	1.04
	*P*.value	**0.02**	0.47	0.62	0.54	0.09	0.29	0.7	0.8	0.63
Estrogene receptor	Positive (27)	0.57	0.76	0.81	0.8	1.06	0.5	0.41	1.53	0.76
	Negative (35)	1.62	1.41	0.68	0.48	1.32	0.14	0.35	0.86	1.54
	*P*.value	**0.0008**	**0.008**	0.95	0.86	0.36	**0.04**	0.57	**0.03**	0.19
Progesterone receptor	Positive (25)	0.59	0.67	0.81	0.5	0.9	0.39	0.37	1.06	0.56
	Negative (37)	1.36	1.56	0.66	0.48	1.53	0.17	0.39	0.97	1.54
	*P*.value	**0.007**	**0.001**	0.53	0.87	0.15	0.25	0.44	**0.03**	0.69
HER2	Positive (27)	0.67	0.79	0.81	0.53	1.06	0.39	0.4	1.16	0.76
	Negative (35)	1.49	1.41	0.66	0.42	1.32	0.17	0.35	0.9	1.54
	*P*.value	**0.02**	**0.03**	0.66	0.54	0.45	0.34	0.73	**0.03**	0.75
Lymph nodes metastasis	Yes (28)	1.42	1.21	0.85	0.58	1.21	0.12	0.29	1.1	1.05
	No (34)	0.63	0.98	0.57	0.34	1.11	0.29	0.49	0.85	1.09
	*P*.value	**0.008**	0.41	**0.03**	0.31	0.65	0.09	0.1	**0.04**	0.9
Histologic grade	G1 (10)	0.29	0.29	0.21	0.17	0.38	0.54	0.25	0.6	0.72
	G2 (33)	1.04	1.29	0.81	0.48	1.06	0.28	0.49	1.16	1.11
	G3 (19)	2	1.56	0.68	0.55	1.62	0.12	0.35	0.9	1.44
	*P*.value	**0.01**	0.06	0.15	0.06	0.28	0.42	0.17	0.93	0.93

^a^The mean expression of all studied genes has been compared between the defined subgroups

## Discussion

Personalized or precision medicine is considered one of the main approaches to improving patients’ treatment efficacy and survival. Molecular investigations have paved the way for the classification and utilization of personalized medicine for different subtypes of BC [26].

BRCAness is a term that describes this phenotype in tumors with HRD [27]. Approximately more than 66% of TNBC show BRCAness, whereas less than 14% of all cases of sporadic BC show this phenotype [7, 27]. It is of great importance to detect BRCAness in BC since it serves as a biomarker for the administration of PARP inhibitors as well as DNA-damaging chemotherapy [28, 29]. However, a major challenge is to find biomarkers that identify BRCAness subgroup—tumors with somatic mutations for HRR genes and/or low levels of HRR-related genes expression—to guide treatment of PARP inhibitors and DNA-damaging chemotherapy. Here, we have investigated the potential expression signatures for BRCAness in sporadic BC.

Dysregulation of genes involved in the HRR pathway could cause BRCAness. Non-coding RNAs have gained more attention in recent years as key regulators of gene expression, and some miRNAs have been demonstrated as a biomarker of BRCAness in sporadic BC [30–32]. Since cell-free miRNAs are stable and detectable in plasma and saliva, they have been recognized as promising minimally invasive biomarkers for diagnosing and prognosis of different diseases [33, 34]. The expression profiling of miRNAs has also revealed novel diagnostic, predictive, and prognostic biomarkers in BC [35–37].

We hypothesized that specific miRNAs targeting genes involved in the HRR induce BRCAness in BC and can be potential biomarkers of BRCAness in plasma. To assess this notion, based on the screening of the GEO dataset, Targetscan, DIANA, and miRTarBase databases, and literature review, we have opted for 3 multi-targeted miRNAs, including miR-182-5p, miR-146a, and miR-498, which are involved in the HRR pathway (Fig. 1). Previous studies showed that miR-182-5p with pro-tumor functions and overexpression in BC is one of the central miRNAs targeting a network of genes in this pathway [38, 39]. It has been shown that miR-182 increases sensitivity to PARP inhibitors. It is also a potential prognostic factor for BC and associates with poor survival [39, 40]. The miR-146a also decreases the expression of BRCA1, causing BRCAness in TNBC [41]. Its role as a prognostic factor associating with improved survival has been previously demonstrated [42]. The miR-498 is another regulator of BRCA1 expression in TNBC [43]. In addition to BRCA1, these miRNAs target other genes encoding essential proteins in the HRR. In the current study, to uncover the dysregulation of pivotal miRNA-mRNAs axes involving HRR, we have opted for 6 downstream genes, including BRCA1, BRCA2, CHEK2, BRIP1, RAD51, and BARD1, for expression assessment (Fig. 1). It seems that the expression assessment of the selected miRNAs and mRNAs would be of great importance to track BRCAness in BC at the transcriptional level.

Based on our results, the expression of miR-182-5p and miR-146a-5p is increased in the breast tumor samples compared to the paired adjacent normal tissues. These results are consistent with previous reports [44, 45]. Moreover, patients with TNBC compared to the non-TNBC subtype displayed increased expression of miR-182-5p and miR-146a-5p. The clinicopathologic analyses revealed that the expression level of miR-182-5p associates with larger tumor size, lymph node metastasis, and higher histologic grade in the BC patients. These results, in accordance with previous reports [46–48], imply the oncogenic roles of miR-182-5p in BC.

Expression analyses of the six key genes in HRR pathway among breast tumors and adjacent normal tissues indicate a significant downregulation of four genes, including BRCA1, CHEK2, RAD51, and BRIP1. This observation confirms the prominent role of HRD in BC progression. Interestingly, when the expression levels of the target genes were compared among TNBC and non-TNBC samples, we observed a significantly decreased expression of BRCA1, CHEK2, and RAD51 in the TNBC samples. Consistent with previous reports, this result emphasizes a higher frequency of BRCAness in TNBC [7, 27, 49]. Since our samples were obtained from patients with sporadic BC, these dysregulations in gene expression could result from somatic mutations or epigenetic alterations. CHEK2 is a tumor suppressor gene whose mutation associates with a moderate risk of hereditary BC [50, 51]. Women with BRIP1 or RAD51 mutations are also at risk of BC [52–54]. Interestingly, BARD1 and RAD51, along with BRCA1 and BRCA2 are susceptibility genes of TNBC [55]. Riaz. et al. demonstrated that somatic pathogenic mutations in CHEK2, RAD51, and BRIP1 genes in breast tumors are extremely rare [56]. So, here we focused on potential dysregulated miRNA/mRNA axes in BC.

In the current study, among all suspected miRNA/mRNA axes (Fig. 1), we found a significant negative correlation among miR-182-5p and two of its targets, CHEK2 and RAD51. This observation suggests the miRNA-mRNA interactions playing roles to induce BRCAness in BC. The molecular interaction of miR-182-5p with CHEK2 has been confirm in previous studies [38, 57]. It should be noted that our results propose the novel miR-182-5p/RAD51 regulatory axis in BC. The 3́UTR of RAD51 mRNA has a conserved site for miR-182-5p seed sequence.

Cell-free analysis of plasma samples from the BC patients showed overexpression of miR-182-5p in 32% of TNBC patients and only in 7.2% of non-TNBC patients (Table 2). Our results indicate that the expression of miR-182-5p in patientś tumor and plasma samples has a positive correlation, although it did not reach statistical significance (*P*. value = 0.058). Therefore, our study suggests that the expression level of cell-free miR-182-5p might be a potential non-invasive biomarker of BRCAness in BC, especially for TNBC patients. This hypothesis should be assessed in a larger sample size. In the previous reports, miR-182-5p has been introduced as an oncomiR in BC by targeting multiple HRR pathway components [38].

## Conclusions

**Fig. 6:**
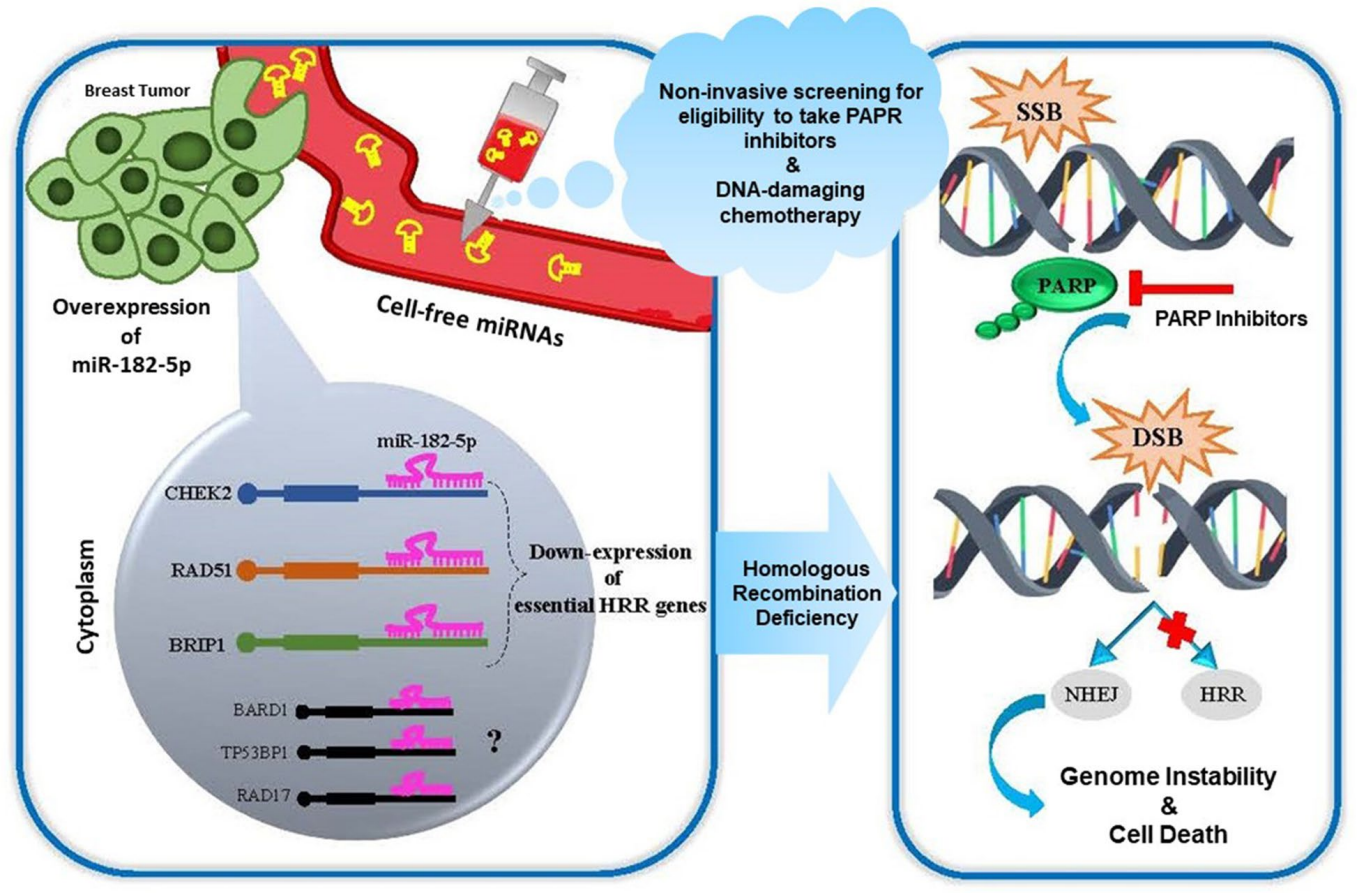
Schematic image of the potential role of miR-182-5p in homologous recombination deficiency. The up-regulation of cell-free miR-182-5p in plasma samples of BC patients could be a non-invasive predictive biomarker for using PARP inhibitor or DNA damaging chemotherapy. The high expression of miR-182-5p could decrease some critical HRR-related genes, such as CHEK2, RAD51. HRR: homologous recombination repair; SSB: single-strand break; DSB: double-strand break; NHEJ: non-homologous end joining; PARP: Poly (ADP-ribose) polymerase

TNBC, as the most challenging subtype of BC with failure to common therapies, requires the administration of precision treatments. Due to the higher frequency of BRCAness in TNBC, the investigation of related biomarkers can assist in predicting response to precision treatments. Here, we highlight the difference between TNBC and non-TNBC in dysregulation of the key miRNA/mRNA axes involved in the HRR pathway. Our study revealed that miR-182-5p is a potential expression signature of BRCAness that associates with decreased expression of pivotal HRR genes, including CHEK2 and RAD51 (Fig. 6). Also, for the first time, we show that the level of cell-free miR-182-5p in BC patientś plasma could be a clue for screening TNBC patients eligible for receiving PARP inhibitors and/or DNA-damaging chemotherapy through a personalized manner. Further evaluation of the expression of miR-182-5p in clinical trials testing PARP inhibitors or DNA-damaging chemotherapy for TNBC can validate its predictive values.

## Supplementary Information

**Additional file 1: Supplementary Table 1.** Differentially expressed (DE) miRNAs in triple negative compared with other breast cancer subtypes (GEO microarray dataset GSE19536). **Supplementary Table 2.** Linear regression analysis for investigating the linear effect of miR-182-5p expression on CHEK2 and RAD51 expression in breast tumors. **Supplementary Table 3.** Spearman correlation between miR-146a-5p and its targets. **Supplementary Table 4.** Spearman correlation between miR-498 and its targets.

**Additional file 2: Supplementary Fig. 1.** Heat map showing expression pattern (log2 transformed) of miR-182-5p, miR-146a-5p, miR-498 and their downstream targets in BC samples and adjacent normal tissues. The expression values are arranged from red (low expression) to blue (high expression). Each row shows one sample, and each column demonstrates a transcript.

## Data Availability

The datasets supporting the conclusions of this article are available in: [GEO dataset] at (https://www.ncbi.nlm.nih.gov/geo/geo2r/?acc=GSE19536&platform=GPL8227). The data supporting the conclusions of this article are also included within the article and supplementary materials.

## Abbreviations

BC: Breast cancer
TNBC: Triple negative breast cancer
HRR: Homologous recombination repair
HRD: Homologous recombination deficiency
miRNA: MicroRNA
mRNA: Messenger RNA
ncRNA: Non-coding RNA
NHEJ: Non-homologous end joining
PARP: Poly (ADP-ribose) polymerase
DSB: Double strand break
SSB: Single strand break
