# Supplementary material for: Investigation of BRCAness associated miRNA-gene axes in breast cancer: cell-free miR-182-5p as a potential expression signature of BRCAness
Source: BMC Cancer. 2022 Jun 17;22:668. doi: 10.1186/s12885-022-09761-4 (PMC9206264; doi:10.1186/s12885-022-09761-4)
Supplement: Supplementary file 2 — Additional file 2: Supplementary Fig. 1. Heat map showing expression pattern (log2 transformed) of miR-182-5p, miR-146a-5p, miR-498 and their downstream targets in BC samples and adjacent normal tissues. The expression values are arranged from red (low expression) to blue (high expression). Each row shows one sample, and each column demonstrates a transcript. [file 12885_2022_9761_MOESM2_ESM.docx]

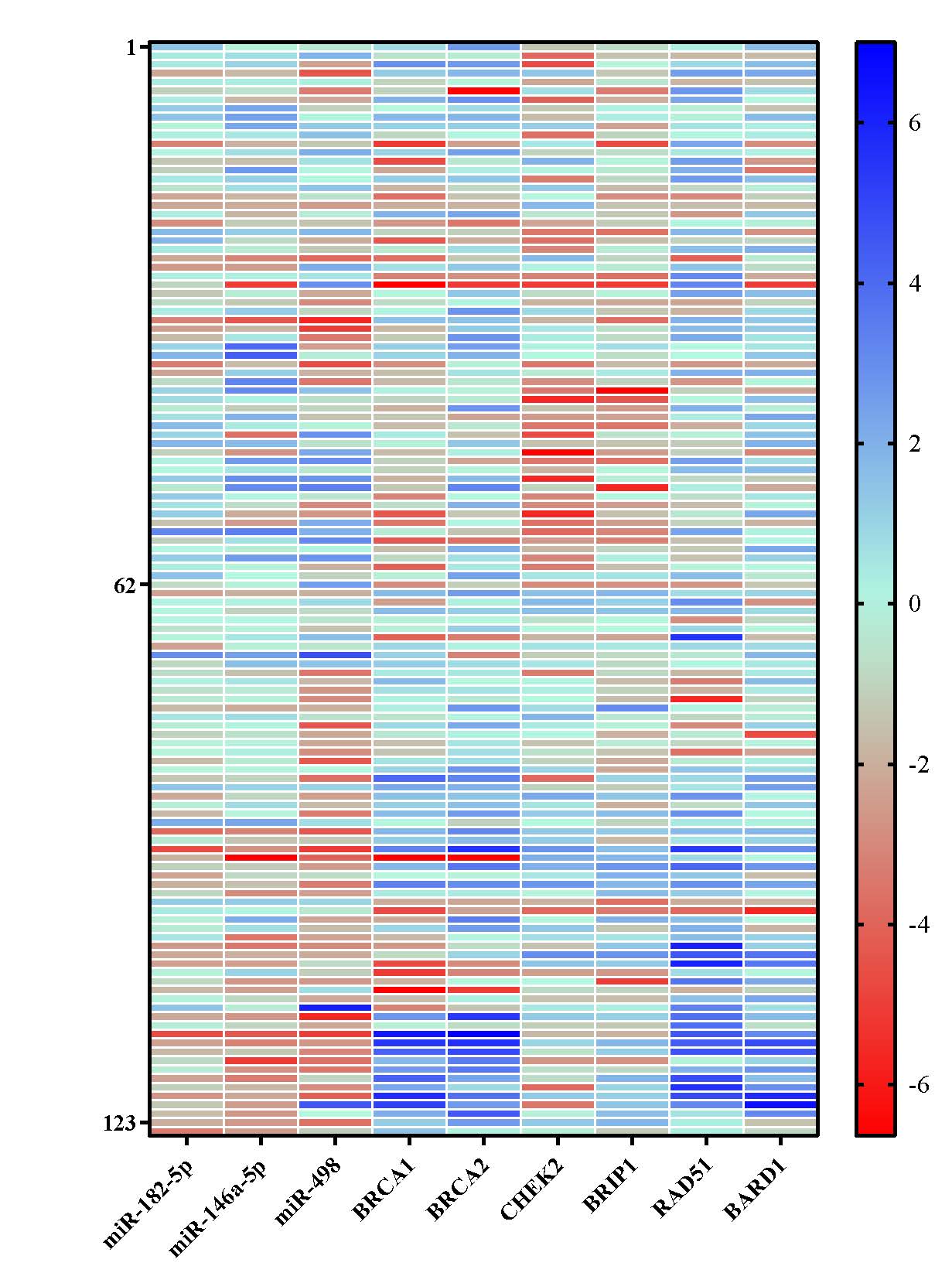


**Supplementary Figure 1.** Heat map showing expression pattern (log2 transformed) of miR-182-5p, miR-146a-5p, miR-498 and their downstream targets in BC samples and adjacent normal tissues. The expression values are arranged from red (low expression) to blue (high expression). Each row shows one sample, and each column demonstrates a transcript.

**Breast tumors**

**Adjacent normal tissues**

**Relative expression (log2 ^fold change)^**
